# Signal quality as Achilles’ heel of graph theory in functional magnetic resonance imaging in multiple sclerosis

**DOI:** 10.1038/s41598-021-86792-0

**Published:** 2021-04-01

**Authors:** Johan Baijot, Stijn Denissen, Lars Costers, Jeroen Gielen, Melissa Cambron, Miguel D’Haeseleer, Marie B. D’hooghe, Anne-Marie Vanbinst, Johan De Mey, Guy Nagels, Jeroen Van Schependom

**Affiliations:** 1 Center For Neurosciences, Vrije Universiteit Brussel, Brussels, Belgium; 2 AZ Sint-Jan, Brugge, Belgium; 3 National MS Center Melsbroek, Melsbroek, Belgium; 4 Department of Radiology, Vrije Universiteit Brussel, Brussels, Belgium; 5 St Edmund Hall, University of Oxford, Oxford, Great Britain and Northern Ireland UK; 6 Department of Electronics and Informatics (ETRO), Vrije Universiteit Brussel, Brussels, Belgium; 7 Ke.2.13; Pleinlaan 2, 1050 Elsene, Belgium

**Keywords:** Multiple sclerosis, Functional magnetic resonance imaging

## Abstract

Graph-theoretical analysis is a novel tool to understand the organisation of the brain.

We assessed whether altered graph theoretical parameters, as observed in multiple sclerosis (MS), reflect pathology-induced restructuring of the brain's functioning or result from a reduced signal quality in functional MRI (fMRI). In a cohort of 49 people with MS and a matched group of 25 healthy subjects (HS), we performed a cognitive evaluation and acquired fMRI. From the fMRI measurement, Pearson correlation-based networks were calculated and graph theoretical parameters reflecting global and local brain organisation were obtained. Additionally, we assessed metrics of scanning quality (signal to noise ratio (SNR)) and fMRI signal quality (temporal SNR and contrast to noise ratio (CNR)). In accordance with the literature, we found that the network parameters were altered in MS compared to HS. However, no significant link was found with cognition. Scanning quality (SNR) did not differ between both cohorts. In contrast, measures of fMRI signal quality were significantly different and explained the observed differences in GTA parameters. Our results suggest that differences in network parameters between MS and HS in fMRI do not reflect a functional reorganisation of the brain, but rather occur due to reduced fMRI signal quality.

## Introduction

The human brain is regarded as a complex architecture of regions, characterized by their unique contribution to brain functioning. Mutual connection between these regions give rise to a complex network. Graph theoretical analysis (GTA) enhances interpretability by reducing its complexity to a limited number of characteristics from a network, namely by representing brain regions and functional connections respectively as the nodes and edges of a simplified network^1^.

GTA can be applied to a wide variety of medical imaging and neurophysiology data. In recent years, this field has gained great popularity and has been proposed as a potential key to unravel the mystery of brain functioning^1,2^. Brain diseases alter the properties of brain networks and understanding these changes offers insight in the nature of the underlying disorders^2,3^.

Since multiple sclerosis (MS) causes damage to the central nervous system by inflammation, demyelination and neurodegeneration^4^, it is hypothesized that damage and response to damage are reflected in the graphs that are derived from brain imaging. Several studies^5–7^ in fact demonstrated a disruption of different network parameters derived from functional Magnetic Resonance Imaging (fMRI) in people with MS (PwMS) compared to healthy subject (HS). GTA has also shed new light on our understanding of cognitive impairment in MS. Network parameters were deviant when comparing cognitively impaired (CI) patients to cognitively preserved (CP) patients, and allowed to categorize MS patients in these groups^3,5^. In general, these findings seemed to indicate a reduced global, but relatively spared local connectivity in MS. Yet some discrepancy exists across studies regarding alterations of network parameters^3^.

The overall purpose of investigating these network parameters is to find a potential new biomarker for cognition in MS. Hence our interest in assessing whether earlier findings truly suffice to be termed a “biomarker”, and thus reflect a genuine change in brain functioning, or by measurement bias in fMRI as evoked by side effects of MS. Since fMRI relies on the blood oxygen level dependent (BOLD) signal to indirectly measure the brain activity, and because of the vulnerability of this method to pathology-induced pitfalls^8^, this study aims to reconsider network alterations in MS in the light of fMRI quality issues.

## Methods

### Compliance with ethical standards

The study was approved by the ethical committee of the University Hospital Brussels (Commissie medische ethiek (O.G. 016) Reflectiegroep Biomedische Ethiek) on the 15th of July 2014 for the protocol B.U.N. 143201421363 and a second ethical approval was obtained on the 25th of February 2015 for the protocol B.U.N. 143201423263. The data analysis was performed according to these study protocols and following the local applicable regulations. A written informed consent of all participants of the study was obtained prior to the measurements.

### Data collection

Fifty PwMS and twenty-six HS were included in this study. Anatomical T1-weighted MR images, resting state fMRI (rsfMRI), cognitive test results and demographical data were acquired for each study participant. PwMS were recruited in the National MS Center of Melsbroek and in the UZ Brussel. The HS were recruited in the community of the PwMS and amongst hospital staff, and were matched for gender, age and education level. All study participants were aged between 18 and 65 years and were able to undergo MRI (absence of contra-indications, e.g. pacemaker, prosthesis). Moreover, only PwMS with an MS diagnosis as defined by the revised McDonald criteria^9^ and an Expanded Disability Status Scale score^10^ (EDSS) lower than 6 were considered. First-degree relatives to a person with MS were excluded as HS.

### Cognitive testing

The neuropsychological test battery contained the Symbol Digit Modalities Test (SDMT, information processing speed), California Verbal Learning Test-II (CVLT-II, verbal memory and learning), Brief Visuospatial Memory Test Revised (BVMT-R, visual memory and learning) and Controlled Oral Word Association Test (COWAT, verbal fluency). The SDMT, CVLT-II and BVMT-R together form the BICAMS test battery^11^, which was validated in Belgium^12^.

### MRI scanning

All scans were done at UZ Brussel on a 3 T Philips Achieva scanner. The sagittal T1 weighted brain MRIs were acquired with the following parameters: field of view: 240 mm × 240 mm and 310slices, voxel size: 0.5 mm × 0.5 mm × 0.5 mm voxels, flip angle: 8°, repetition time (TR): 5.19 s, echo time (TE): 2.30 s. The rsfMRI’s were acquired with eyes closed while staying awake. Following parameters were used: field of view: 230 mm × 230 mm and 48 slices, voxel size: 1.8 mm × 1.8 mm × 2.7 mm voxels, flip angle: 90°, TR: 3 s, TE: 35 s.

### Pre-processing

The pre-processing of the images was performed using MATLAB 2017a and SPM12^13^. The different steps and their sequence were based on the technical papers on fMRI pre-processing from Weissenbach et al., Liang et al. and Jo et al.^14–16^ The first and last ten timeframes were removed to avoid any transition or start-up phenomenon. The images were realigned, and patients requiring an excessive correction above 1 mm translational or 0.0125 radians rotational deformation were discarded to ensure measurement of a quasi-stationary subject^17,18^. Slice timing correction was applied and rigid-body co-registration to the T1 image was performed for each subject individually. Once individual co-registrations between the fMRI and the T1 were obtained, anatomical variations between persons needed to be compensated. This was achieved by estimating the deformation field from the T1 segmentation in comparison to the tissue probability maps in Montreal Neurological Institute (MNI) space from SPM12^13^ for grey and white matter, cerebrospinal fluid, skull or bone tissue and soft tissue. For each subject, an fMRI sequence in MNI space was interpolated based on the inverse of the obtained deformation field and the corresponding co-registered fMRI. Spatial smoothing was not applied as we used a parcellation atlas^19^. Subsequently, only extracranial signals were used to correct for background noise^14,20–22^, as an alternative to global signal regression. The latter is suboptimal since it removes a part of the neuronal signal^20^.

### Signal extraction to network parameters

Next, the averaged time course was extracted and bandpass filtered between 0.009 Hz and 0.08 Hz^14^ for each of the 94 cortical regions as defined in the AAL2 parcellation atlas^23^. Each of these regions was considered as a node in our network. All nodes are interconnected through edges. The weights of these edges were determined by calculating the Pearson's correlation between the signals of the respective regions and were subsequently orderly grouped in an adjacency matrix. We used a threshold on the edge weight varying from 0 to the highest value before the network splits in disconnected subnetworks, and calculated the following GTA parameters on the different binarized networks: degree for the amount of connections, clustering coefficient and transitivity to describe interconnectivity of the graph, shortest path length, local efficiency and global efficiency to quantify the ease of information transfer through the network, and small-worldness to understand the influence of the network topology on communication within the network^1,24^.

### fMRI data quality

**Figure 1:**
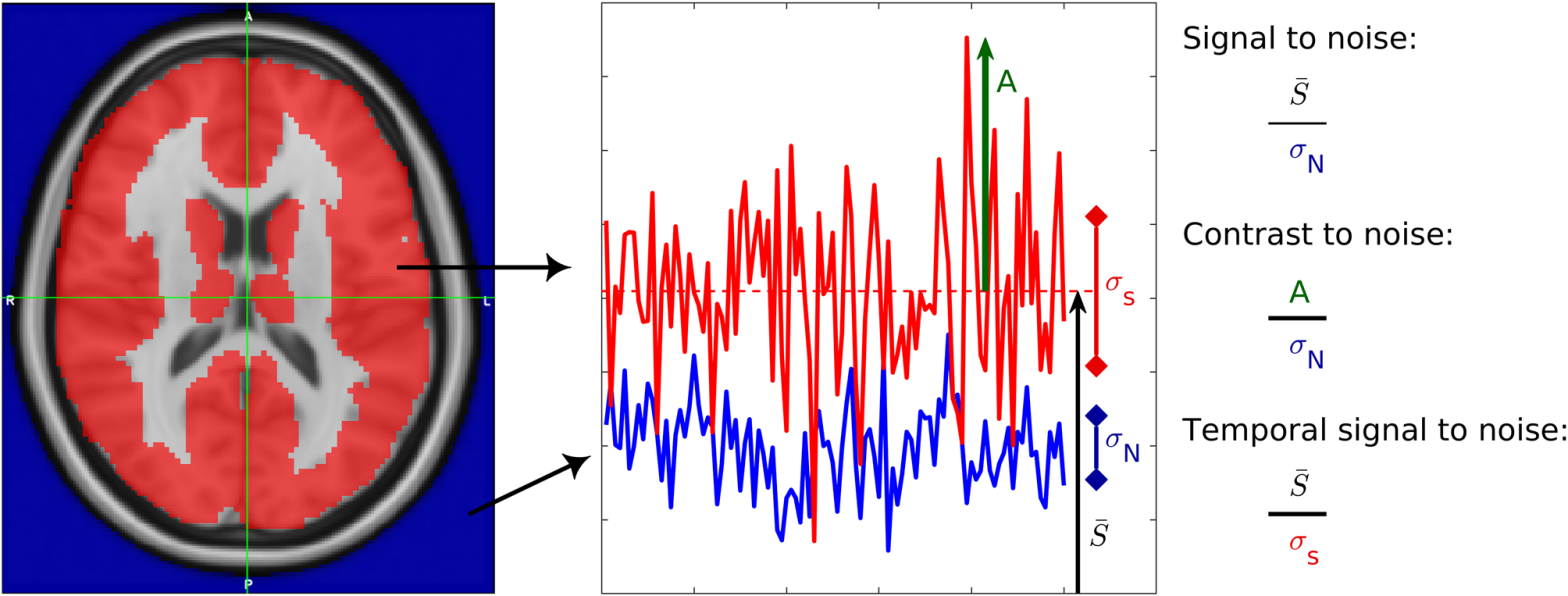
Illustration of the fMRI quality metrics. The red and blue signal represent brain activity and background noise respectively. $\overline{S}$ is the mean of the brain activity, **σ**_**S**_ is the standard deviation of the brain activity, **σ**_**N**_ is the standard deviation of the background noise, and **A** is the amplitude of the maximum peak of the brain activity.

An fMRI is a sequence of fast acquired MRI images. A distinction is made between the measured signal, which is the magnetic recovery over time of an excited region/voxel, and the signal of interest, reflecting the fluctuation of the measured signal in a region. Quality metrics of fMRI include measures of scanning quality (signal to noise ratio (SNR)) and signal quality (temporal SNR (tSNR) and contrast to noise ratio (CNR)). All three parameters are explained both visually (Fig. 1) and narratively (cfr infra).

The SNR is a measure of scanning quality of the individual images which together form the fMRI, namely the mean fMRI signal divided by the standard deviation of the background noise. The background noise is evaluated by measuring the scanned voxels of the region where no anatomical matter is present. This has been done using the probability map available in SPM12 for air or background to select the correct voxels^25^.

The tSNR is the mean fMRI signal divided by its standard deviation and shows overtime signal variation. It therefore assesses the quality of the fMRI signal^25^.

The CNR is the amplitude of the signal (absolute value of maximum signal peak minus signal average) divided by the standard deviation of the noise. This parameter also assesses the quality of the fMRI signal, in this case by quantifying the detectability of the contrast of interest^25^.

The data quality parameters are evaluated on the fMRI images in standard space (after pre-processing).

### Graph analysis

At every threshold, the network parameters are calculated for every subject. A linear regression on the obtained network parameters was performed for age and education level. The network parameters of PwMS were compared to HS using permutation tests of 10,000 permutations. To establish if there is a link between brain changes caused by MS and cognitive deterioration, the Pearson’s correlations between all seven studied GTA parameters and the seven cognitive parameters (SDMT, CVLT, BVMT, COWAT, FSMC, BICAMS scaled score and BICAMS z-score) were evaluated. Correction for multiple comparison was performed with a familywise error correction method. We used the method of Dunn-Šidák^26^. This was repeated including CNR and tSNR as covariates in the model.

Second, the entire process was repeated including CNR and tSNR in the regression equation to assess their impact on GTA parameters. Moreover, their contributions to the equation were compared between PwMS and HS using 10,000 permutations.

## Results

### Study population

We included 50 PwMS and 26 HS. One of each group was discarded due to motion during scanning. The characteristics and cognitive test results of the remaining PwMS and HS are presented in Table 1. PwMS and HS differed with respect to fatigue and depression, but were similar with respect to age, level of education and gender distribution. HS scored significantly higher on both verbal and visual memory as tested by CVLT-II and BVMT-R respectively.

**Table 1:** Demographics and cognitive scores of the PwMS and HS group. P-values were generally derived from two-sample t-tests, with the exception of a Chi-square test for age. (y: years; IQR: Interquartile range).

	PwMS (n = 49)	HS (n = 25)	p-value
Age in y (Mean ± SD)	44.7 (± 11.1)	41.6 (± 12.7)	0.28
Gender (Men/Women)	6/43	5/20	0.38
Education level in y (Mean ± SD)	14.2 (± 2.8)	14.8 (± 2.4)	0.53
Beck’s Depression Inventory (Mean ± SD)	12.2 (± 9.0)	5.8 (± 4.8)	**0.0017**
Fatigue Scale for Motor and Cognitive Functions (Mean ± SD)	63.8 (± 18.2)	37.6 (± 10.0)	** < 0.0001**
Expanded disability status Scale (Median [IQR])	2.5 [1–3]	–	–
Disease duration in y (Mean ± SD)	10.9 (± 8.1)	–	–
Cognitive evaluation:			
SDMT (Mean ± SD)	53.7 (± 12.3)	53.6 (± 9.0)	0.98
CVLT-II (Mean ± SD)	61.4 (± 11.1)	68.1 (± 6.3)	**0.007**
BVMT-R (Mean ± SD)	25.9 (± 6.7)	29.6 (± 4.2)	**0.013**
COWAT (Mean ± SD)	32.2 (± 11.1)	35.0 (± 9.8)	0.21

p-values below the statistical threshold of significance (0.05) are indicated in bold.

### GTA group differences

**Figure 2:**
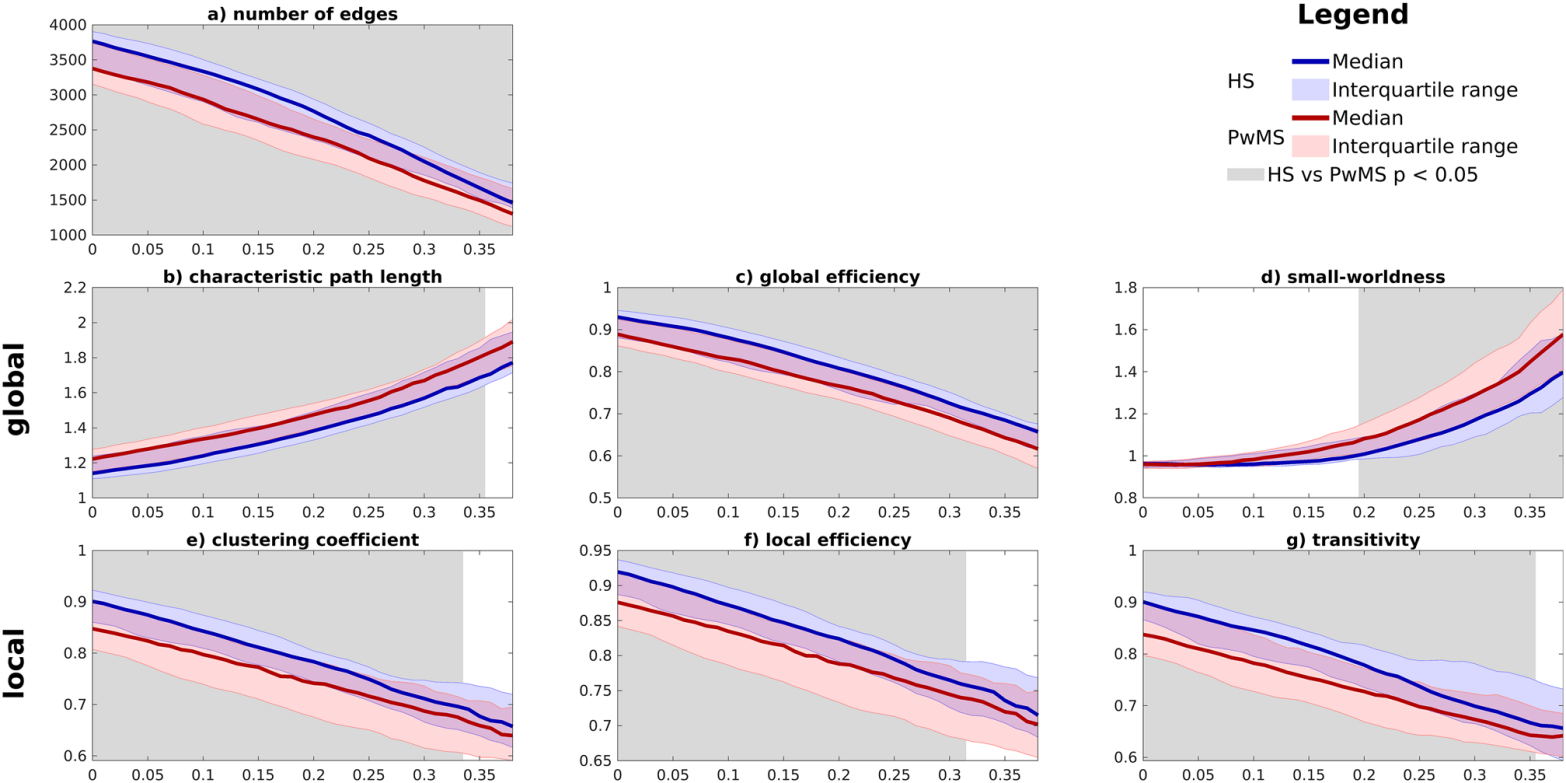
Graphs (**a**) to (**d**) show the global network parameters and (**e**) to (**g**) show the local network parameters. The median value and interquartile range of PwMS (red) and HS (blue) are presented. The x-axis represents the thresholds from the adjacency matrix from which networks were extracted. The background is shown in grey for the regions where the permutation test between the two groups showed a significant difference (p < 0.05).

All pre-processing steps were checked for outliers and similarity between all patients and both groups. Since the first disconnected network occurred at a threshold of 0.39, we evaluated the earlier discussed GTA parameters for thresholds varying from 0 to 0.38. The results are presented in Fig. 2. All network parameters show differences between PwMS and HS. Number of edges, global efficiency, clustering coefficient local efficiency and transitivity were significantly smaller in MS versus HS in the entire considered threshold range. The characteristic path length was significantly larger in MS within the same range. Small-worldness did not differ between MS and HS for thresholds below 0.2. Small-worldness was larger in MS above of the threshold of 0.2.

### Correlation with cognition

We evaluated the Pearson’s correlation between all GTA parameters for every cut-off and all elements of the neuropsychological test battery. However, none of the correlations were statistically significant We observed at best p = 0.008 for CVLT and global efficiency, which does not survive correction for multiple comparisons.

### Adjacency matrix and MS model

**Figure 3:**
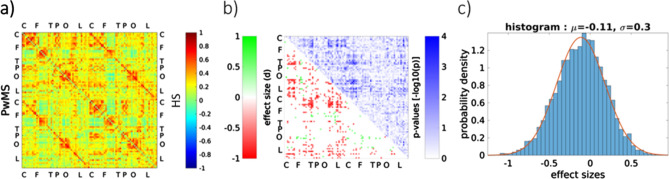
(**a**) the adjacency matrix of HS in the upper triangle matrix and PwMS in the lower triangle matrix. (**b**) results of the permutation tests between all edges, PwMS relative to HS (**c**) distribution of all effect sizes of the permutations with p-value < 0.05.

When observing the edges individually, as shown in Fig. 3, no edge significantly differs between HS and PwMS after correction for multiple comparisons. A trend towards a general lowered functional connectivity (FC) is visible especially between the edges connecting ipsilateral central/frontal and lateral/temporal regions. In HS, the overall mean FC is 0.27, compared to 0.23 in PwMS.

### SNR, CNR and tSNR

**Figure 4:**
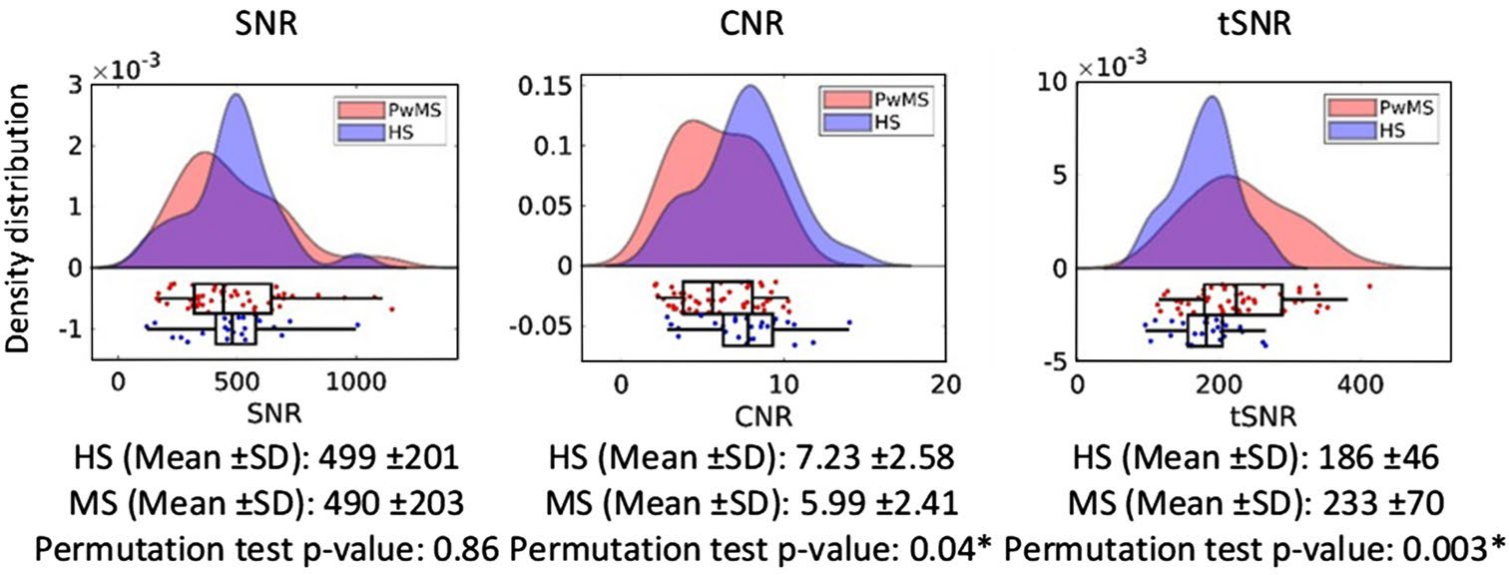
Group distributions of scanning quality (SNR) and signal quality (tSNR and CNR) of the fMRI scans in standard space. * indicates statistical significance (p < 0.05).

As shown in Fig. 4, the CNR is smaller (p = 0.04) and the tSNR larger (p = 0.003) in MS compared to HS. We did not find any difference in SNR. More details about the data quality components are available in supplementary Table 1.

### Group differences in GTA parameters after correction for fMRI signal quality

**Figure 5:**
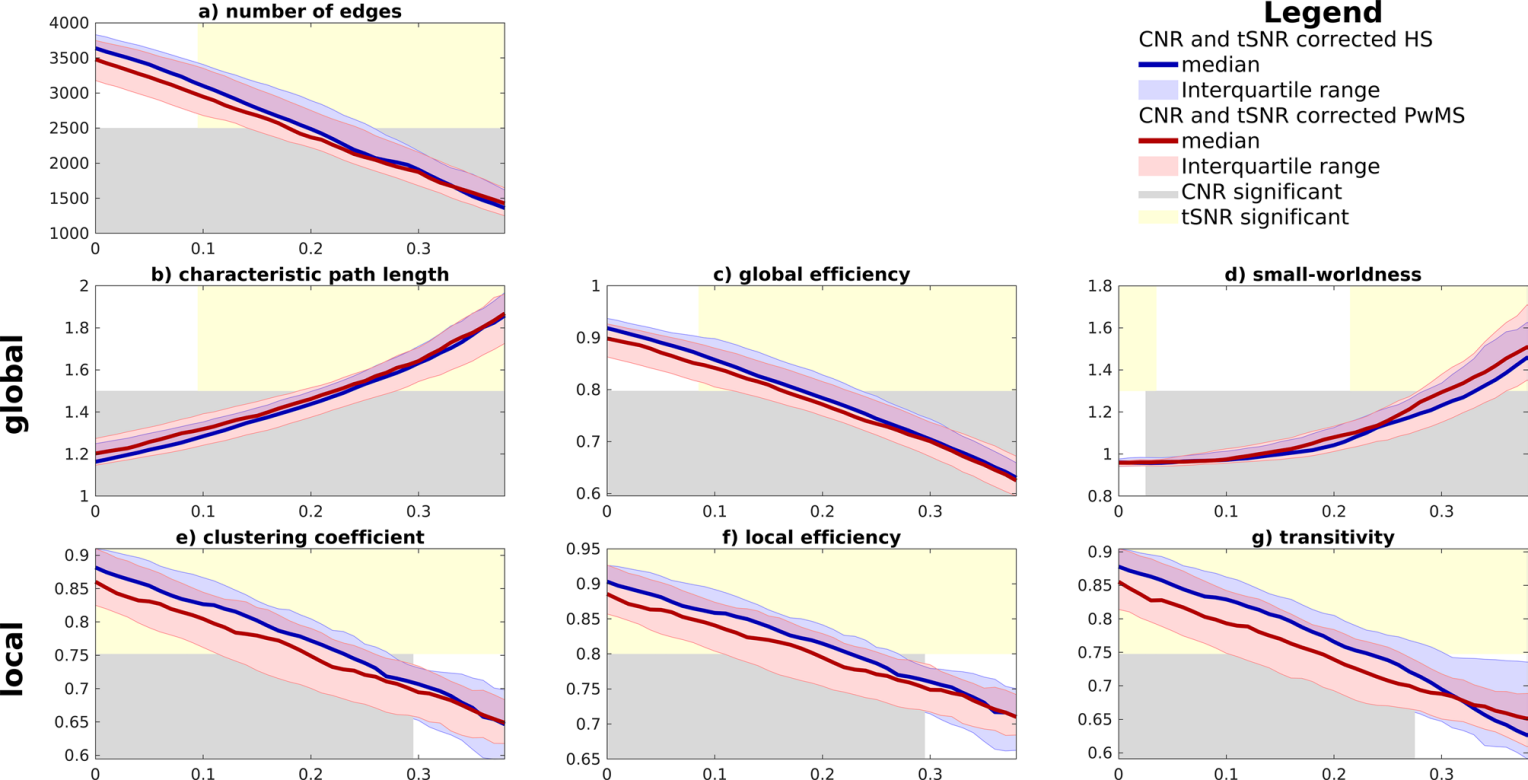
Graphs (**a**) to (**d**) show the global network parameters and e to g show the local network parameters. The median value and interquartile range of the GTA parameters, corrected for CNR and tSNR, are presented for PwMS (red) and HS (blue). The x-axis represents the thresholds from the adjacency matrix from which networks were extracted. The background is shown in grey and yellow for CNR and tSNR respectively to indicate at what thresholds they contributed significantly.

After linear correction of the GTA-parameters for tSNR and CNR, differences between both populations completely disappeared. Figure 5 also shows when CNR and tSNR contributed significantly to the linear regression.

## Discussion

In this study, we assessed whether changes in GTA parameters could be explained by fMRI signal quality, rather than pathology. Before correcting for differences in fMRI signal quality, our data show differences in GTA parameters in MS with respect to HS, comparable to those reported in previous literature^3,5,6^. Once we corrected for fMRI signal quality by including CNR and tSNR as covariates in our analyses, the observed differences disappeared.

Our network analysis before correction showed a decrease in both global and local connectivity in the MS group. However, after thresholding, the number of remaining edges in PwMS was lower than in HS which is a direct result of the apparent diffuse decrease in edge weights. This means that after thresholding, we are comparing networks with different numbers of edges between PwMS and HS, biasing all the measured GTA parameters. The network extraction method is of paramount importance in the interpretation of the difference in mean connectivity. This was underlined by adopting an alternative method of network extraction whereby equal network sizes are assured, thus enhancing comparability. Networks extracted by the latter method do not show differences in GTA parameters.

### Underlying causes of data quality differences

The apparent reduction in FC influencing network parameters is reflected by a significantly increased tSNR. This increase is caused by both a (non-significant) increase in the mean signal and a (non-significant) decrease in signal variation. Post-hoc analysis revealed that the data quality parameters did not differ when calculated in subject space and a voxel-wise comparison indicated that increases in tSNR occurred predominantly in the vicinity of the cerebral ventricular system. This indicates that standard registration procedures as applied in this study and many others^5–7^ may not be suitable to analyse fMRI data in patients with neurodegenerative diseases. Additionally, we looked at the different available co-registration steps within SPM. For a more elaborate visual and narrative explanation, we refer to the supplementary data, although it is justified to mention here that varying the registration and normalisation parameters in SPM resulted in similar periventricular discrepancies. However, there are two causes that may affect signal quality. First, we used a non-linear co-registration method (SPM Lesion Normalization with Tissue Probability Maps^13^ as used before^5,27^ and recommended^14,28^). This algorithm can cause a general inflation of voxels, as smaller MS brains are co-registered to the same standard MNI space as non-atrophied healthy brains.

Secondly, the presence of lesions in the MS population. While most MS patients have some lesions or atrophy around the ventricles, cortical lesions are more diffusely spread across the cortex (without preferential location). This explains why signal quality is most obviously altered around the ventricles and not over the full cortex. Unfortunately, cortical lesions cannot be measured through the acquired scans and a double inversion recovery sequence is not available for these patients. Lesion filling can to some extent be used to counter these effects^28,29^. We did not apply lesion filling in our study as we wanted to be able to compare our results to previously published papers on graph theoretical measures in MS patients^5–7,27^.

Phantom studies reveal that tSNR is inversely correlated to dynamic fidelity which is the degree to which fMRI captures the true BOLD fluctuations^30^. Thus, the observed decreased tSNR in the control group indicates an increased capability to capture BOLD fluctuations in comparison to MS.

The decrease in CNR is plausibly explained by a reduced vascular coupling in MS^8,31^. The reduced blood flow^26^, will generate a slower replenishment of the metabolic demand and thus a diminished BOLD signal and lower signal peak^8^. This hypothesis is further strengthened by the fact that noise levels were comparable between both groups. More specifically, a second post-hoc analysis showed no significant difference in motion of subjects within the scanner (translation and rotation) between PwMS and HS.

Regarding SNR, we did not find a significant difference between groups. This is not surprising since SNR strongly depends on hardware and scanning sequence used for the fMRI acquisition, being identical for all subjects in our study. The observed individual variations in SNR depend on anatomical variations between subjects and variations in the background noise at the time of measurement^25^.

Lastly, as neural activation could also influence the functional connectivity, we performed a third post-hoc analysis to compare the amplitude of low-frequency fluctuations, defined as the power between 0.01 and 0.08 Hz^32^ between PwMS and HS. However, no differences in ALFF could be observed (data shown in the supplementary material).

In summary, we hypothesize that a reduced vascular coupling and registrational issues of individual MS brain images from subject space to MNI space underly our observed differences in signal quality between PwMS and HS.

### How signal quality affects functional connectivity and thus GTA parameters

Both a higher tSNR (r = 0.3, p = 0.0045) and CNR (r = -0.43, p = 0.00013) are correlated with a higher FC and as such affect all GTA metrics they summarise the network’s properties^1^. Whereas most papers interpret changes in GTA measures as expressions of a neurological reorganization, our results suggest that the origin lies in differences in signal quality between MS and HS. This is further corroborated by simulations (see supplementary material) that clearly demonstrate that lower CNR and higher tSNR are accompanied by lower correlation.

### Extension of results to other neurodegenerative pathologies

The differences in GTA parameters between PwMS and HS that were initially observed, disappeared after correcting for fMRI signal quality. This means that alterations in GTA parameters in MS are driven by fMRI signal quality and should not be appointed to cerebral reorganizational processes. This potentially extends to other neurodegenerative pathologies leading to cognitive impairment, such as frontotemporal dementia (FTD) and Alzheimer’s disease (AD). In FTD, cognitive problems were associated with increased characteristic path length, decreased global efficiency and clustering coefficient, and a decreased average network degree^33^, which reflects lowered connectivity. These findings are in accordance with MS literature. In AD, fMRI studies suggested a decrement in small-worldness and diminished global organisation^34–36^. These studies used a different method to extract networks, namely by using a proportional method that fixed the number of edges. However, this approach does not guarantee that connections of equal strengths are compared^1^, again obfuscating the comparison.

### Limitations

Some limitations should be mentioned regarding the adopted methodology. First, when extracting the networks, only one linear bivariate connectivity measure (Pearson’s correlation) was considered. Another connectivity measure (e.g. Granger Causality^37^ and mutual information^38^) might reveal a more complex relation that has now been overlooked. Nonetheless, these methods likely suffer from the same registration issues. Second, we focussed on the effect of fMRI signal quality on graph theory in MS, not the underlying cause of this quality decrement. An arterial spin labelling study^31^, further research on the BOLD signal^8^ could offer valuable insight. Third, we did not account for the influence of pharmacological treatment, possibly altering brain perfusion and brain activity. Some drug treatments impact the neurovascular coupling generating the BOLD signal^8^. Due to heterogeneity in type and dose of medication among the PwMS included in our study, we were unable to perform a sensitivity analysis taking this additional variable into account.

### Recommendations for best practice

GTA parameters are sophisticated analytical parameters to summarize characteristics of networks, for example the brain. Changes in these parameters will often depend on how the network was built, implying cautious interpretation of their relationship with clinical symptoms like cognitive disturbances in MS.

FMRI indirectly measures brain activity, and complex disease activity in MS, like atrophy or cardiovascular disturbances, can bias its interpretation. By compensating for fMRI signal quality metrics (CNR and tSNR), this could be mitigated.

## Conclusion

In conclusion, this study confirmed changes in graph-theoretical measures in multiple sclerosis patients in accordance with previously reported results. However, we demonstrate that these changes may result from decreased fMRI signal quality, rather than pointing towards a functional reorganization of the brain. Hence, fMRI results obtained in MS should be interpreted carefully.

## Supplementary Information

Supplementary Information

## Data Availability

Data sharing is not possible due to privacy and ethical restrictions.
