# Supplementary material for: Signal quality as Achilles’ heel of graph theory in functional magnetic resonance imaging in multiple sclerosis
Source: Sci Rep. 2021 Apr 1;11:7376. doi: 10.1038/s41598-021-86792-0 (PMC8016888; doi:10.1038/s41598-021-86792-0)
Supplement: Supplementary file 1 — Supplementary Information [file 41598_2021_86792_MOESM1_ESM.docx]

Signal quality as Achilles’ heel of graph theory in functional magnetic resonance imaging in multiple sclerosis

Johan Baijot^1†^, Stijn Denissen^1†^, Lars Costers^1^, Jeroen Gielen^1^, Melissa Cambron^1,2^, Miguel D'Haeseleer^1,3^, Marie B D'hooghe^1,3^, Anne-Marie Vanbinst^1^, Johan De Mey^4^, Guy Nagels^1,3,5‡^, Jeroen Van Schependom^1,4,6‡^,

1 Vrije Universiteit Brussel, Center For Neurosciences, Brussels, Belgium

2 AZ Sint-Jan, Brugge, Belgium

3 National MS Center Melsbroek, Melsbroek, Belgium

4 Vrije Universiteit Brussel, Department of Radiology, Brussels, Belgium

5 St Edmund Hall, University of Oxford, Oxford, United Kingdom of Great Britain and Northern Ireland

6 Vrije Universiteit Brussel, Department of Electronics and Informatics (ETRO), Brussels, Belgium

† Baijot J. and Denissen S. should be considered joint first author.

‡ Van Schependom J. and Nagels G. should be considered joint senior author.

Corresponding author: Johan Baijot ✆ +322/629 29 39 🖂 [johan.baijot@vub.be](mailto:johan.baijot@vub.be)
🖃 Ke.2.13; Pleinlaan 2; 1050 Elsene, Belgium
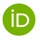
 https://orcid.org/0000-0003-1108-7845

# Supplementary material

## Individual components of the data quality

Supplementary Table 1. Comparison of individual parameters of the signal quality (HS vs PwMS). P-values were derived from permutation tests with HS (n=25) and PwMS (n=49). Values are presented as mean ± std and the * denotes a statistical significant p-value (p<0.05).

|  | **HS** | **PwMS** | **p-value** |
| --- | --- | --- | --- |
| **SNR** | 499 ± 201 | 490 ± 203 | 0.86 |
| **CNR** | 7.23 ± 2.58 | 5.99 ± 2.41 | **0.04*** |
| **tSNR** | 186 ± 46 | 233 ± 70 | **0.003*** |
| $\bar{\boldsymbol{S}}$ | 118 ± 27 | 124 ± 26 | 0.42 |
| **σ_n_** | 0.29 ± 0.19 | 0.31 ± 0.18 | 0.59 |
| **A** | 1.75 ± 0.56 | 1.61 ± 0.65 | 0.40 |
| **σ_s_** | 0.61 ± 0.15 | 0.57 ± 0.23 | 0.43 |

## Post-Hoc analysis: Data quality parameters in subject space

To obtain the image quality factors in subject space we determined the brain and the region scanned outside the subject head for noise estimation. The T1-image was co-registered to the fMRI images and segmented to obtain a mask of the grey matter. To obtain the scanned region outside the brain we used the MNI template and corresponding mask of the head which we co-registered to each individual subject. We note that in subject space, we encounter inter-subject variability in brain volume. From supplementary Figure 1 we can conclude that image quality measurements in subject space were comparable between HS and PwMS.


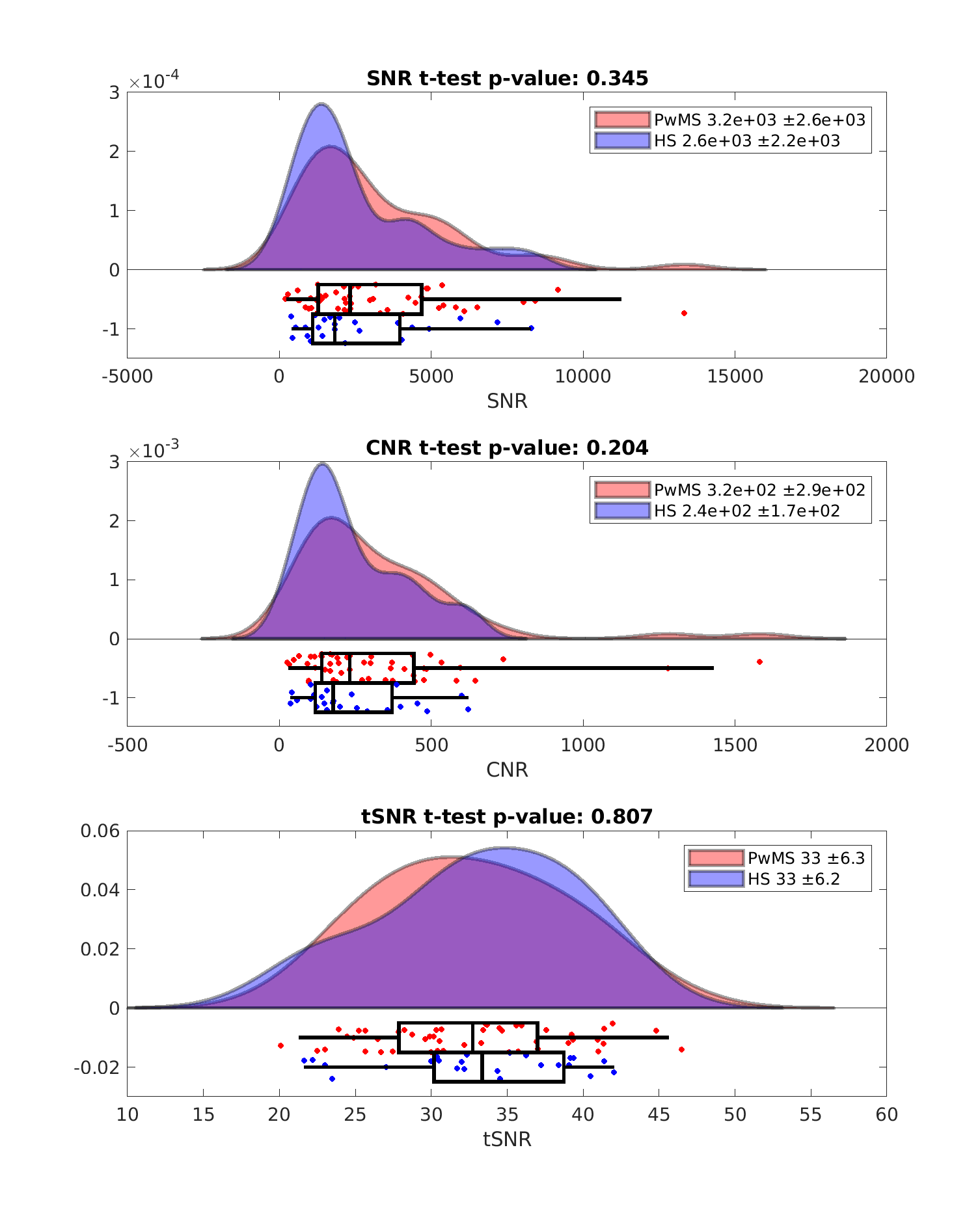


Supplementary Figure 1: Data quality parameters in subject space. The variables are shown using raincloud plots which combine a histogram with a boxplot view of the data for each group. HS are shown in blue and PwMS in red. The data in the legend is denoted as mean ± standard deviation.

## Post-Hoc analysis: co-registration

In a post-hoc analysis we looked at possible variations of the pre-processing steps. In particular, different co-registration algorithms, available within SPM12^1^, were assessed. tSNR was calculated in a voxel-wise way, and t-tests were used to compare them between PwMS and HS. As illustrated in Supplementary Figure 2, discrepancies were mostly present around the ventricles. The normalised cross-correlation co-registration method was discarded as this method did not result in a correct co-registration, presenting a clear offset between the images upon visual inspection.


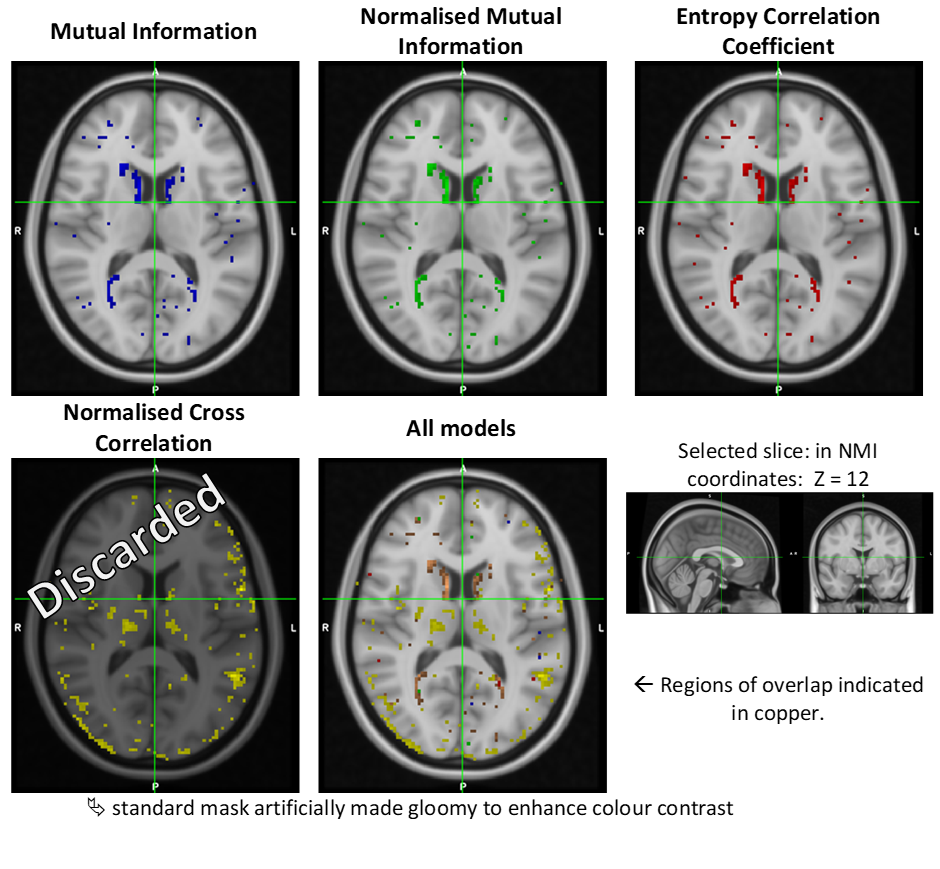


Supplementary Figure 2: Post-hoc comparison of SPM12 co-registration models voxel-wise comparison between the tSNR of HS and PwMS.

## Amplitude of low frequency fluctuations (ALFF):

In a secondary post-hic analysis We have calculated the ALFF, defined as the power between 0.01Hz and 0.08Hz^2^ for each of the study participants. As ALFF has also been linked to neural activity, We performed two analyses to see if our previous analysis overlooked any neural activity visible with ALFF:

### Voxelwise comparison of ALFF between HS and PwMS

For every voxel, we ran a permutation test (N=10000) comparing ALFF in healthy subjects (HS) to the ALFF in people with multiple sclerosis (PwMS). Next, we corrected for multiple comparisons using the method of Dunn- Šidák.^3^ As depicted in Figure 3, the ALFF differs significantly (p<0.05) between both cohorts, but importantly, no differences survive correction for multiple comparisons.


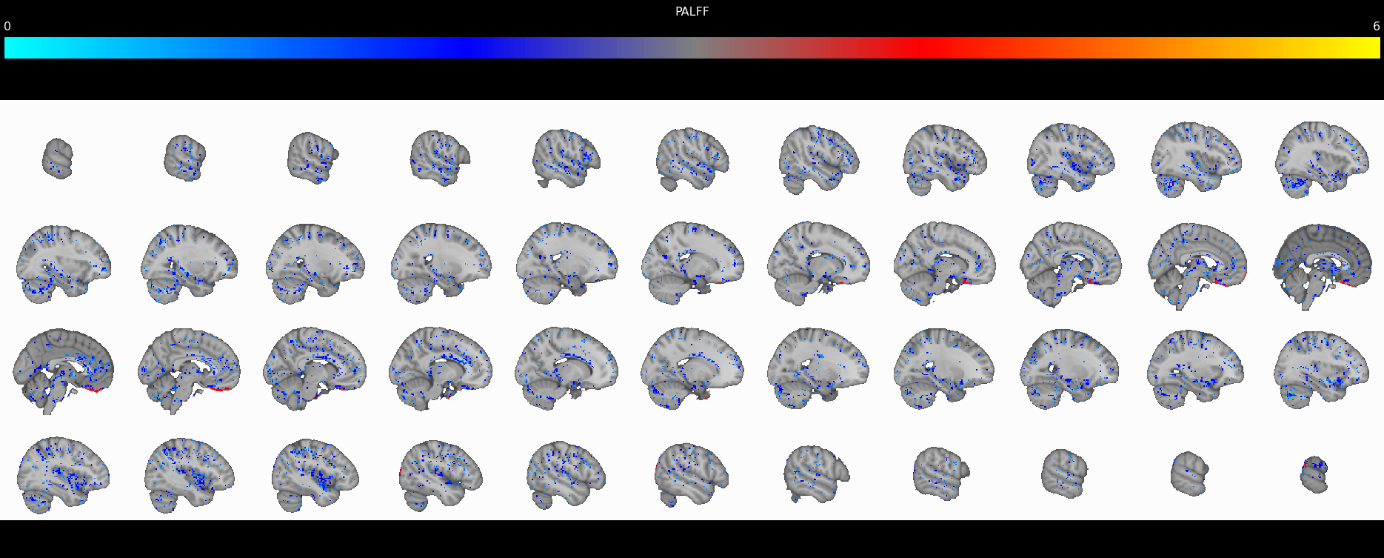


Colour bar: 0
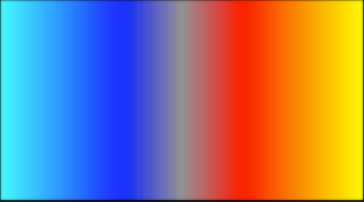
 6

Supplementary Figure 3: Voxel-wise comparison of ALFF between HS and PwMS, without correction for multiple comparisons. The colour indicates the p-value yielded from permutation tests (10.000 permutations). The p-value is transformed with the inverse logarithmic scale.

### Voxelwise correlation between ALFF and SNR, CNR and tSNR

In addition to the comparison MS vs HS, we also calculated the Pearson correlation between the ALFF and the 3 different quality parameters. Again, no significant relationships were observed when correcting for multiple comparisons. Figure 4 provides visual support, but without correcting for multiple comparisons.

|  | **SNR** | **CNR** | **tSNR** |
| --- | --- | --- | --- |
| **Rho** | 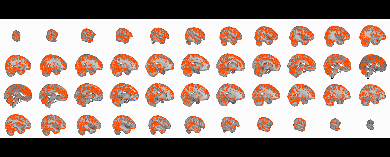 | 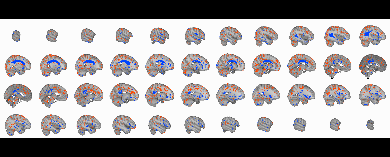 | 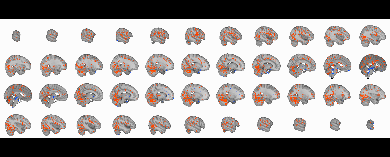 |
|  | Colour bar: -1 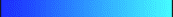 0 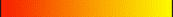 +1 | | |
| **-log10(p)** | 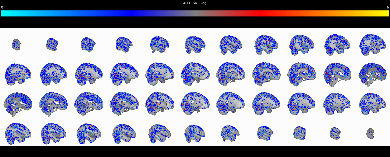 | 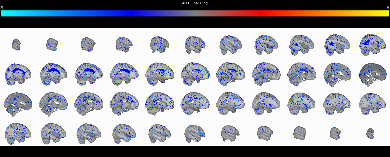 | 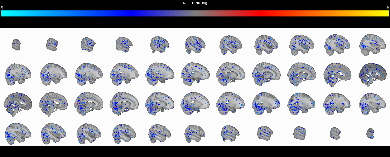 |
|  | Colour bar: 0 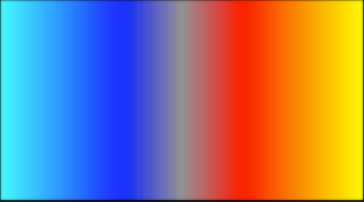 6 | | |

Supplementary Figure 4**:** Correlation between ALFF and the 3 data quality parameters using the Pearson correlation, without correcting for multiple comparisons. The top row shows the strength and direction of the correlations, the bottom row shows the significance of these correlations. The p-value is transformed to the inverse logarithmic scale and an initial cut-off of 0.05 was applied (-log10(0.05) = 1.3).

In this additional analyses, we do not observe any ALFF differences between HS and PwMS and no significant correlation with data quality parameters.

### How signal quality affects functional connectivity additional simulation:

To provide further insight on how signal quality affects the calculation of FC, we present the following additional analysis, which consists of 2 parts:

1. **Real data**. We calculated the Pearson correlation between FC on the one hand, and either CNR or tSNR on the other. Both correlations were statistically significant, with an r = 0.3 (p = 0.0045) for CNR, and r = -0.43 (p = 0.00013) for tSNR. This is visible in the top 2 figures of supplementary figure 5.
2. **Simulated data**. We simulated 500 pairs of signals y_i,1_ and y_i,2_, which can be interpreted as simulated versions of the real BOLD signals, using the following equations (where i ranges from 1 to 500):

$$y_{i,0,CNR}=120+0.23*CNR*{rand}_{uniform \left[ -1\to1 \right]}(t)$$

$$y_{i,0,tSNR}=120+\left( \frac{120}{tSNR} \right)*{rand}_{Gaussian}(t)$$

$$[y_{i,1};y_{i,2}]=[y_{i,0};y_{i,0}]*Chol(0.27)+backgroundnoise(t,i)$$

- We used an offset of 120, because this was the observed offset in our data.
- 0.23 is the average variance of the background noise in our data
- CNR was varied between 1 and 15 in steps of 0.2 in correspondence with the observed CNR values in our data.
- ${rand}_{uniform [-1\to1]}$ is a uniform random distribution between -1 and 1
- tSNR was varied between 50 and 400 in correspondence with the observed tSNR values in our data.
- ${rand}_{Gaussian}$ is Gaussian noise
- Chol is the Cholesky decomposition of the desired correlation matrix for a set correlation.
- The set correlation was chosen as 0.27, because this was the effective average correlation (FC) from our study.
- The background noise is Gaussian noise which simulates the background noise in the scanner which had a mean of 0 and a variance of 0.23 (average variance of the background noise in our data).

Subsequently, we calculated the correlation between the two simulated signals y_i,1_ and y_i,2_ (y axis in bottom 2 figures in Supplementary figure 5), with varying values of CNR and tSNR (x axis).

In the bottom figures, we observe that a lower CNR and higher tSNR accompanies a decreased approximation of the 0.27 benchmark. This provides direct evidence that both tSNR and CNR affect the true FC. For tSNR, this finding is consistent with earlier findings by Golestani et al.^4^


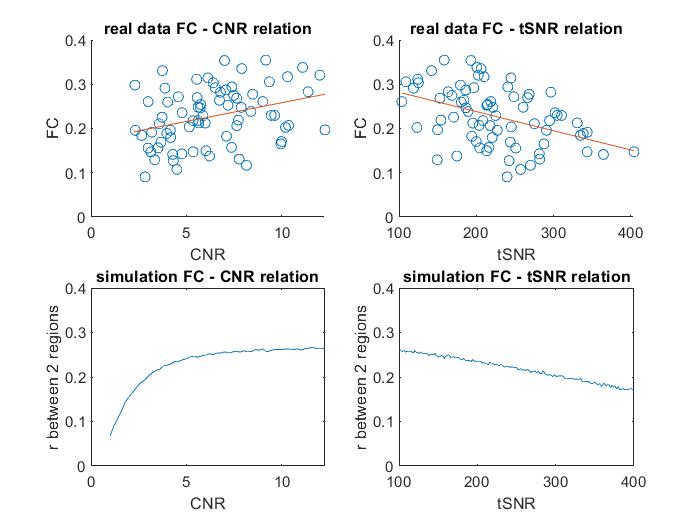


Supplementary Figure 5: The top figures represent real data used in our study, and shows a significant relationship between signal quality (CNR and tSNR) and FC. The bottom two figures represent results of the simulations. The x axes equal those of the top figures (real data), while the y axis represent the Pearson correlation between 2 regions, of which the signals were simulated.

## References:

1. Penny, W. D., Friston, J. K., Ashburner, T. J., Kiebel, J. S. & Nichols, E. T. *Statistical parametric mapping: the analysis of functional brain images: the analysis of functional brain images*. *Functional neuroimaging: Technical* (Elsevier, 2011).

2. Liu, Y. *et al.* Brain plasticity in relapsing-remitting multiple sclerosis: Evidence from resting-state fMRI. *J. Neurol. Sci.* **304**, 127–131 (2011).

3. Sidák, Z. Rectangular Confidence Regions for the Means of Multivariate Normal Distributions. *J. Am. Stat. Assoc.* **62**, 626–633 (1967).

4. Golestani, A. M. & Goodyear, B. G. A Resting-State Connectivity Metric Independent of Temporal Signal-to-Noise Ratio and Signal Amplitude. *Brain Connect.* **1**, 159–167 (2011).
